# The inhibitory effects of metabolites from *Bacillus pumilus* on potato virus Y and the induction of early response genes in *Nicotiana tabacum*

**DOI:** 10.1186/s13568-020-01089-1

**Published:** 2020-08-20

**Authors:** Shuo Shen, Wei Li

**Affiliations:** 1 Guizhou University, Guiyang, 550025 Guizhou China; 2 Academy of Agriculture and Forestry Sciences, Qinghai University, 810016, Xining, Qinghai China; 3 Key Laboratory of Potato Breeding of Qinghai Province, Xining, 810016 Qinghai China; 4 State Key Laboratory of Plateau Ecology and Agriculture, Xining, 810016 Qinghai China; 5 The Tibet Plateau Biotechnology Key Lab of Ministry of Education, Xining, 810016 Qinghai China

**Keywords:** Halophilic bacterium *Bacillus pumilus*, Active compounds, Inhibitory activity, *Potato Virus Y* genes encoding viral proteins, Early response genes

## Abstract

To develop a new antiviral preparation from a microbial source, the halophilic bacterium *Bacillus pumilus* E303035 was isolated from a soil sample collected at Qarhan Salt Lake in Qinghai, China. The inhibitory activity of an ethyl acetate extract of its fermentation broth was higher than that of an n-butanol extract. After isolation and purification, 9 compounds were obtained: cyclo(L-Leu-L-Pro) (**1**), cyclo(L-Pro-L-Tyr) (**2**), Brevianamide F (**3**), 2-(3-Indolyl) ethanol (**4**), N-[2-(1H-indol-3-yl) ethyl] acetamide (**5**), 3, 3-di(1H-indol-3-yl)propane-1,2-diol (**6**), Lincomycin B (**7**), dibutylphthalate (**8**), and p-hydroxyphenethyl alcohol (**9**). Compounds **1**, **5**, and **9** showed inhibitory activities against potato virus Y (PVY). Compounds **1**, **4**, and **9** had significant inhibitory activity against genes *HC*-*pro*, *P3*, and *Nib*, compound **5** against gene *P3*, and compounds **1** and **4** against *NIa*. Compounds **1**, **4**, **5**, and **9** had significant inhibitory activity against genes *VPg* and *6K1*. Active compounds **1**, **5**, and **9** had various effects on the expression of viral genes related to pathogenesis. Expression of genes *cullin* and *XTH* was up-regulated and *CP* was down-regulated, compared to the positive control. In conclusion, compounds **1**, **5**, and **9** might be considered as potential antiviral agents for future development.

## Introduction

The species *Potato virus Y* (PVY) is the most important viral pathogen affecting potato crops and a serious threat to potato production worldwide (Davie et al. 2017; Funke et al. 2017). It can infect various crops, including potato, tobacco, tomato, pepper and other crops belonging to the *Solanaceae* family, and can cause serious damage and significant economic loss (Zhang et al. 2017). Currently, few agents are available to effectively control this plant virus, such as ningnanmycin, dufulin, and ribavirin (Xiao et al. 2015). The search for new antiviral compounds is critical, by considering new sources and novel antiviral mechanisms.

Microbes are a broad and rich resource of natural bioactive products. Bacteria of the genus *Bacillus* are known to produce a wide range of active substances. They have successfully been used to generate compounds such as amylase, proteases, antibiotics, and surfactants (Caetano et al. 2011). The antagonistic effects of *Bacillus* are due to secondary metabolite production of antibiotics and antimicrobial peptides (Hu et al. 2010).

In this study, a halophilic *Bacillus* strain *Bacillus pumilus* E303035 with antiviral activity against PVY which we have screened in our lab was chosen. 9 compounds were isolated from this strain. The antiviral activities against PVY of fermentation extracts and isolated compounds were evaluated with real-time PCR. In order to illustrate the inhibitory mechanisms of active compounds isolated from strain E303035, the effects were determined on total RNA and on genes encoding the functional proteins of PVY.

## Materials and methods

### Plant and PVY materials

Tobacco plants (*Nicotiana tabacum* var. Samsun NN) were grown under greenhouse conditions, with 16 h light at 25 °C and 8 h darkness at 15 °C, and PVY was kept at 4 °C, in the Key Laboratory of Qinghai-Tibet Plateau Biotechnology (KLB), Ministry of Education, Qinghai Province, China. Three or four fully expanded leaves from, approximately, one-month-old plants were used in the experiment.

### Strains and preparation of fermentation broth

Halophilic bacterium *Bacillus pumilus* Strain E303035 (Genbank accession number: MN238693; GDMCC number:60077) was isolated from a Qarhan Salt Lake soil sample, and was preserved at 4 °C in the KLB. It was cultured at 37 °C, with a rotation speed of 200 r/min in a shaker cabinet for 7 days, in Erlenmeyer flasks (1 L). Each flask contained 600 mL of ATCC213 medium (10 g MgSO_4_·7H_2_O, 0.2 g CaCl_2_·2H_2_O 0.2 g, KCl, 2.5 g peptone, 10 g yeast extract, 30 g NaCl, adding deuterium-depleted water (D.D.W) to 1000 mL, pH 7.2–7.4).

Then, the fermentation broth was centrifuged at a rotation speed of 8000 r/min, the supernatant was filtered through a 0.22 µm Millipore filter and stored at 4 °C for the preparation of crude extracts (Zhang et al. 2017).

### Preparation of crude extracts of fermentation broth of strain E303035

The fermentation broth of strain E303035 was extracted with either ethyl acetate or n-butanol. The two kinds of extract were concentrated in vacuo to yield a residue. The residues were dissolved in distilled water (using DMSO as a vehicle, at a maximum concentration of 0.1%) to prepare concentrations of 1, 5, 10, and 20 mg/ml for antiviral testing against PVY (Zhang et al. 2017).

### Isolation and identification of compounds in strain E303035

1H, 13C, DEPT, 1H-1H-COSY, HSQC, and HMBC NMR spectra were obtained with a JNM-ECA600 spectrometer. FAB-MS spectra were recorded on a JEOL JMS-HX 110 instrument. The chromatographic stationary phases were silica gel (200–300 mesh), Sephadex LH-20 (25–100 µm, Pharmacia) and MCI-gel CHP20P (75–150 mm, Mitsubishi Chemical). Using thin-layer chromatography, compounds were visualized by spraying with 5% H_2_SO_4_, followed by heating (Shen et al. 2013).

The ethyl acetate extract was dissolved in ethanol and concentrated in vacuo to yield 11 g oily fraction. Afterwards, the fraction was subjected to handera-SI (10 µm), and eluted with *n*-hexane: isopropanol in the range 80:20−50:50, to give Fraction 1 (Fr1) 4.5 g, Fr2 248 mg, Fr3 516 mg, Fr4 597 mg, and Fr5 267 mg.

Fr1 was subjected to MCI-gel CHP20P and eluted with 100% MeOH, MeOH:H_2_O (1:2), and 100% acetone to give compound **8** (3900 mg). Fr2 was subjected to Handera C18 (10 µm) and eluted with C_2_H_3_N-H_2_O (1:10–3:10) to give compounds **4** (51 mg) and **9** (10 mg). Fr3 was subjected to Handera C18 (10 µm) and eluted with C_2_H_3_N:H_2_O (1:20–7:20) to give compound **7** (13 mg). Fr4 was subjected to Handera C18 (10 µm) and eluted with C_2_H_3_N:H_2_O (1:20–3:10) to give compounds **1** (86 mg), **5** (78 mg), and **6** (8 mg). Fr5 was subjected to Handera C18 (10 µm) and eluted with C_2_H_3_N:H_2_O (1:20–3:10) to give compounds **2** (25 mg) and **3** (11 mg).

Compounds **1**–**9** were dissolved in distilled water (using DMSO as a vehicle, at a maximum concentration of 0.1%) to prepare the 31, 63, 125, 250, and 500 mg/ml concentrations for antiviral testing against PVY (Zhang et al. 2017).

### PVY inoculation

Inoculation with PVY, and with PVY with an equal volume of extract (crude extracts, compounds, or ningnanmycin), was conducted on two top leaves of the tobacco plants, using a conventional friction method with a 10× concentration of inoculums. Phosphate buffer inoculation was used as a control. The two infected leaves were collected from each plant, for RNA extraction, 7 days from when the symptoms appeared (Chen et al. 2017).

### Real-time PCR (qPCR) analysis

The total RNA was extracted using a Total RNA Reagent Kit (Takara) from the leaves of plants that were subjected to PVY infection after treatment with each extract (at concentrations of 1, 5, 10, 20 mg/mL, using DMSO as vehicle at a maximum concentration of 0.1%) or compound (at concentrations of 31, 63, 125, 250, and 500 μg/mL, using DMSO as a vehicle at maximum concentration of 0.1%), in triplicate. Total RNA samples were reverse transcribed using a PrimeScriptTMRT reagent Kit with gDNA Eraser (Takara). Real-time PCR was performed with SYBR Premix Ex Taq Kit (Takara), with *β*-*actin* as an internal control. The final volume of the PCR mixture was 20 µL, including 10 µL of the SYBR Green Master mix reagent, 7.8 µL of sterile water, 0.4 µL of DyeII, 1 µL of cDNA, and 0.4 µL (2 mM) of each real-time PCR primer. Primers used were R*β*-*actin* (5´-AAGGGATGCGAGGATGGA-3´) and F*β*-*actin* (5´-CAAGGAAATCACCGCTTTGG-3´), R*PVY* (5´-TTCATCTCCATCCATCATAACC-3´) and F*PVY* (5´-TACAACTTGCATACGACATAGG-3´), R*Lhc* (5´-TTAAGAGAAGAAGCCGAATGTG-3´)and F*Lhc* (5´-CCACACTTCAACTTGCTGAG-3´), R*PSii* (5´- TTTCTCCTCCCTCCCTTTCTCT-3´) and F*PSii* (5´-TTGCTTGACCGTCGTTGTG-3´), R*XTH* (5´-GCGAGGATTTGAGGCACAG-3´) and F*XTH* (5´-GCAACGAGAGGTGGATTAGAGAA-3´), R*CPIP3* (5´-GTCAGTACAGCCAGAGCCAGAA-3´) and F*CPIP3* (5´-AAGCGACTACCAAAACACACACA-3´), R*cullin*-*1* (5´-GTCGCAGAATGTGGCAAGAA-3´)and F*cullin*-*1* (5´-AGAGAAGAGAGATGTGGTTGGT TTG-3´). The following conditions were used: 95 °C for 10 s, 95 °C for 50 s, and 35 cycles of both 95 °C for 6 s and 62 °C for 35 s. Standard curves were constructed using a series of five tenfold dilutions of the cDNA template. The relative expression levels were calculated (Shen et al. 2018).

### PCR analysis

The total RNA was extracted using a Total RNA Reagent Kit (Takara) from the leaves of plants that were subjected to PVY infection after the different treatments of compounds (at the concentrations of 31, 63, 125, 250, and 500 μg/mL, in triplicate. Total RNA samples were reverse transcribed using a PrimeScriptTMRT reagent Kit with gDNA Eraser (Takara). β-actin was used as an internal control. The final volume of the PCR mixture was 20 µL, including 10 µL of 2× FastTaq PCR Master Mix, 1µL of cDNA, 8.6 µL of sterile water, and 0.2µL (2 mM) of each RT-PCR primer. Primers used were R*β*-*actin* (5´-AAGGGATGCGAGGATGGA-3´) and F*β*-*actin* (5´-CAAGGAAATCACCGCTTTGG-3´), R*HC*-*Pro* (5´-ACCAACTCTATAGTGCTTAATGTCAGA-3´)and F*HC*-*Pro* (5´-GGAGTTCTAGACTCAATGGTTCAGT-3´), R*P1* (5´-TTGTGTAACCTTGGAACGCGC-3´) and F *P1* (5´-ATGGCAACTTACATGTCAACGATTC-3´), R*P3* (5´-CTGATGCCGCACATTATATTCTTC-3´) and F*P3* (5´-GGTATTCCTGGGCATGTCCTG-3´), R*NIa* (5´-TTGCTCTACAACAACATCATGATCAA-3´) and F*NIa* (5´-GCCAAATCACTCATGAGAGGTTTAA-3´), R*NIb* (5´-TTGATGGTGCACTTCATAAGTATCG-3´) and F*NIb* (5´-GCTAAACATTCTGCGTGGATGTAT-3´), R*6K1* (5´-CTGGTGTTTAACTTCATGATCCATT-3´)and F*6K1* (5´-CGCTCCACACCAGGTGTTAG-3´), R*VPg* (5´-TTCATGCTCCACTTCCTGTTTTG-3´)and F*VPg* (5´-GGCAAGAACAAATCCAAAAGAATTC-3´), R*CP* (5´-CAGTTCTTGACTCCAAGTAGAGTATG-3´)and F*CP* (5´-GGAAATGACACAATCGATGCAG-3´) (Shen et al. 2018).

### Statistical analysis

The EC_50_ values were determined from concentration-effect curves by linear regression analysis. Statistical analysis was performed using SPSS 20.0, and data were presented as the arithmetic mean ± standard deviation.

## Results

### Antiviral activities of crude extracts against PVY

As shown in Table 1, both ethyl acetate and n-butanol extracts from strain E303035 showed significant antiviral activity against PVY. The inhibitory activity of ethyl acetate extract was higher than that of n-butanol. The inhibition rates of both extracts were concentration-dependent. The ethyl acetate extract possessed its highest inhibitory activity at 10 mg/mL, with an inhibition rate of 99.70%. The n-butanol extract possessed its highest inhibitory activity at 20 mg/mL, with an inhibition rate of 96.43%. The ethyl acetate extract was then isolated to obtain compounds with anti-viral activity against PVY.

**Table 1:** The inhibitory activity of two different extracts from E303035 fermentation against PVY

Concentration (mg/mL)	Inhibition rate(%)	
	Ethyl acetate	n-Butanol
1	85.59 ± 1.66^b^	41.81 ± 7.89^c^
5	94.59 ± 2.03^a^	88.89 ± 0.00^b^
10	99.70 ± 0.12^a^	91.55 ± 10.51^a^
20	99.04 ± 0.17^a^	96.43 ± 1.86^a^

Data are expressed as mean ± SD from experiments with three replicates. Means with different superscript letters a, b, and c in the same column are significantly different, by Duncan’s multiple range test (P < 0.05)

### Isolation of compounds from the halophilic bacterium Bacillus pumilus

**Fig. 1:**
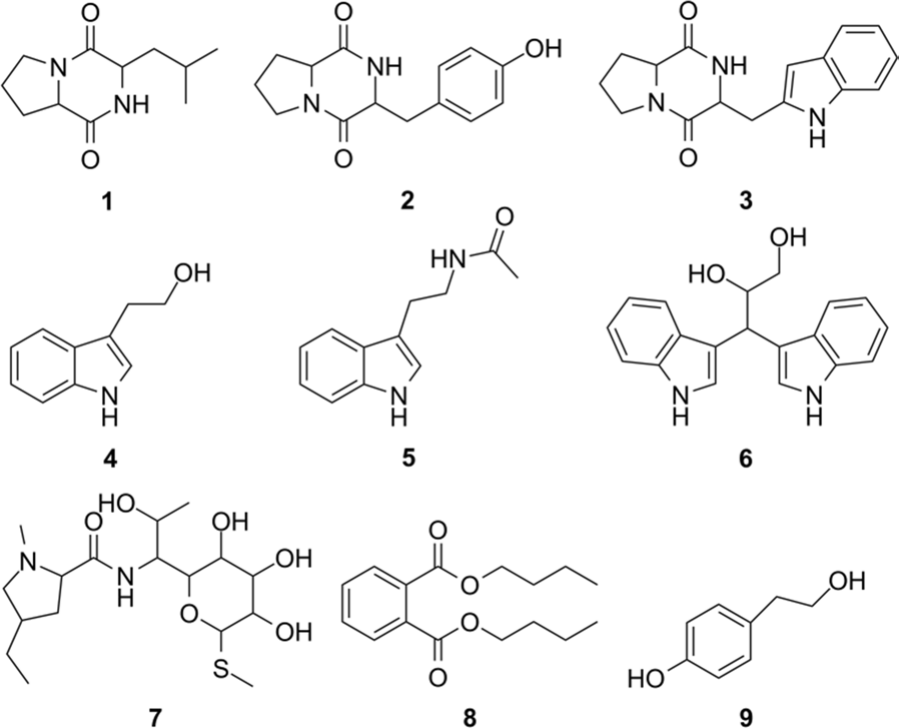
Structures of compounds **1**–**9**

As shown in Fig. 1, 9 compounds were isolated from the ethyl acetate extract of strain E303035 and identified as cyclo(L-Leu-L-Pro) (**1**) (Yue et al. 2010), cyclo(L-Pro-L-Tyr) (**2**) (Li et al. 2013), Brevianamide F (**3**) (Wang et al. 2015), 2-(3-Indolyl) ethanol (**4**) (Böhlendorf et al. 1996), N-[2-(1H-indol-3-yl) ethyl] acetamide (**5**) (Anouhe et al. 2015), 3, 3-di(1H-indol-3-yl)propane- 1,2-diol (**6**) (Zhao et al. 2009), Lincomycin B (**7**) (Argoudelis et al. 1965), dibutylphthalate (**8**) (Dai et al. 2006), and *p*-hydroxyphenethyl alcohol (**9**) (Lou et al. 2001).

*Cyclo*(*L*-*Leu*-*L*-*Pro*) (**1**). Yield: 86 mg; white amorphous powder; molecular formula C_11_H_18_N_2_O_2_.

*Cyclo*(*L*-*Pro*-*L*-*Tyr*) (**2**). Yield: 25 mg; colorless oil; molecular formula C_14_H_16_N_2_O_3_.

*Brevianamide F* (**3**). Yield: 11 mg; white powder; molecular formula C_16_H_17_N_3_O_2_.

*2*-(*3*-*indolyl*) *ethanol* (**4**). Yield: 51 mg; white powder; molecular formula C_10_H_11_NO.

*N*-[2-(1*H*-*indol*-3-*yl*) *ethyl*] *acetamide* (**5**). Yield: 78 mg; yellowish-brown oil; molecular formula C_12_H_13_N_2_O.

*3, 3*-*di*(*1H*-*indol*-*3*-*yl*)*propane*- 1,2-*diol* (**6**). Yield: 8 mg; white powder; molecular formula C_18_H_18_N_2_O_2_.

*Lincomycin B* (**7**). Yield: 13 mg; white powder; molecular formula C_17_H_32_N_2_O_6_S.

*Dibutylphthalate* (**8**). Yield: 3900 mg; brownish red oil; molecular formula C_16_H_22_O_4_.

*P*-*hydroxyphenethyl alcohol* (**9**). Yield: 10 mg; colorless needle crystal; molecular formula C_8_H_10_O_2_.

The data of ^1^H NMR and ^13^C NMR of compounds **1**–**9** is in Additional file 1.

### Antiviral activities of compounds isolated from strain E303035 against PVY

The antiviral activities, against PVY, of compounds isolated from strain E303035 were determined at 31, 63, 125, 250, and 500 μg/mL. As shown in Table 2, compounds **1**, **5**, and **9** possessed higher antiviral activity against PVY, with EC_50_ values of 210.99, 224.26, and 305.37 μg/mL, respectively. Compound **5** showed significant antiviral activity against PVY at 250 and 500 μg/mL, with inhibition values of 62.40% and 75.99%, respectively. Compound **1** also showed moderate antiviral activity at a concentration of 125 μg/mL, with an inhibition value of 53.87%. Compound **4** showed weak antiviral activity at 250 and 500 μg/mL, with inhibition values of 30.21% and 24.29%, respectively. In summary, we found that compounds **1**, **5**, and **9** had good inhibitory activity against PVY, and compounds **1**, **4**, **5**, and **9** were then used for further research of inhibitory activity against genes encoding functional proteins of PVY.

**Table 2:** The antiviral activity of compounds isolated from strain E303035 against PVY

Compound	Inhibition rate( %)					EC_50_ (μg/mL)
	500 (μg/mL)	250 (μg/mL)	125 (μg/mL)	63 (μg/mL)	31 (μg/mL)	
**1**	65.48 ± 1.98^b^	59.12 ± 2.43^b^	53.87 ± 2.47^a^	48.24 ± 2.17^a^	17.06 ± 1.37^b^	210.99
**2**	-*	–	–	–	–	–
**3**	9.68 ± 0.45^d^	–	–	–	–	–
**4**	30.21 ± 2.29^c^	24.29 ± 3.49^d^	–	–	–	–
**5**	75.99 ± 0.54^a^	62.40 ± 1.28^a^	32.34 ± 1.09^c^	31.67 ± 2.33^b^	31.53 ± 2.15^a^	224.26
**6**	–	–	–	–	–	–
**7**	–	–	–	–	–	–
**8**	–	–	–	–	–	–
**9**	61.75 ± 0.62^b^	52.62 ± 3.34^c^	45.78 ± 1.15^b^	28.51 ± 0.46^c^	10.21 ± 2.29^c^	305.37

Data are expressed as mean ± SD from experiments with three replicates. Means with different letters a, b, and c in the same column are significantly different, by Duncan’s multiple range test (P < 0.05). * No inhibitory activity

### Effects of active compounds on the expression of genes encoding functional proteins

**Fig. 2:**
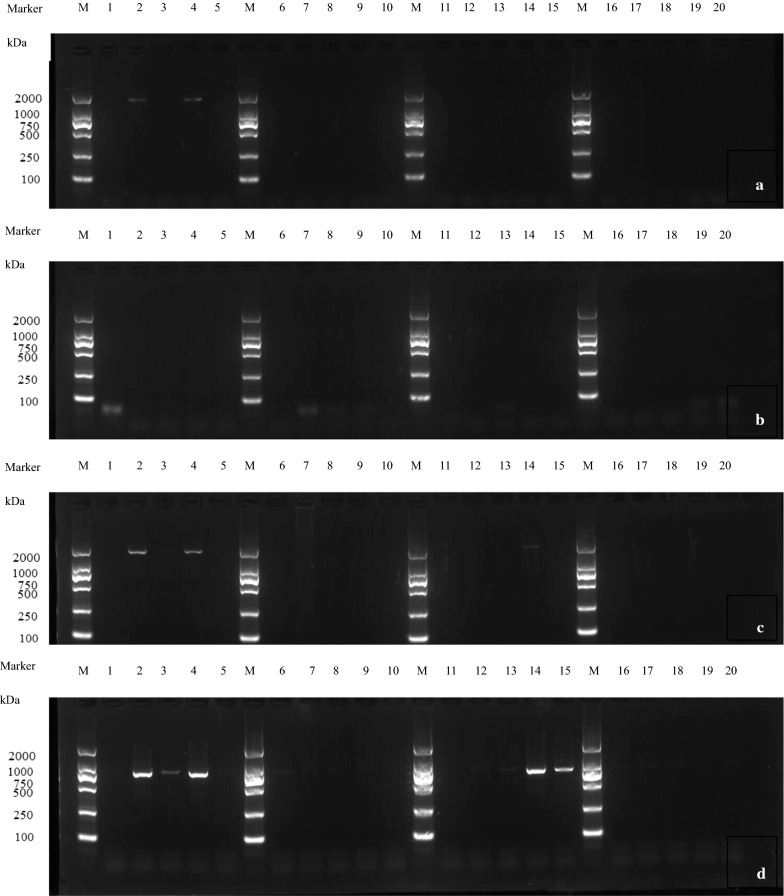
Effect of active compounds on expression of genes encoding PVY functional proteins: *HC*-*pro*, *P3*, *NIb* and *NIa*. **a** gene *HC*-*Pro;* **b** gene *P3;* **c** gene *NIb;* **d** gene *NIa.* M: Marker lane; Series of Lanes 1–5: compound **5**; 6–10: compound **1;** 11–15: compound **9**; 16–20: compound **4.** Each series of 5 lanes between markers represents the concentrations used: 500, 250, 125, 63, and 31 μg/mL, from left to right

**Fig. 3:**
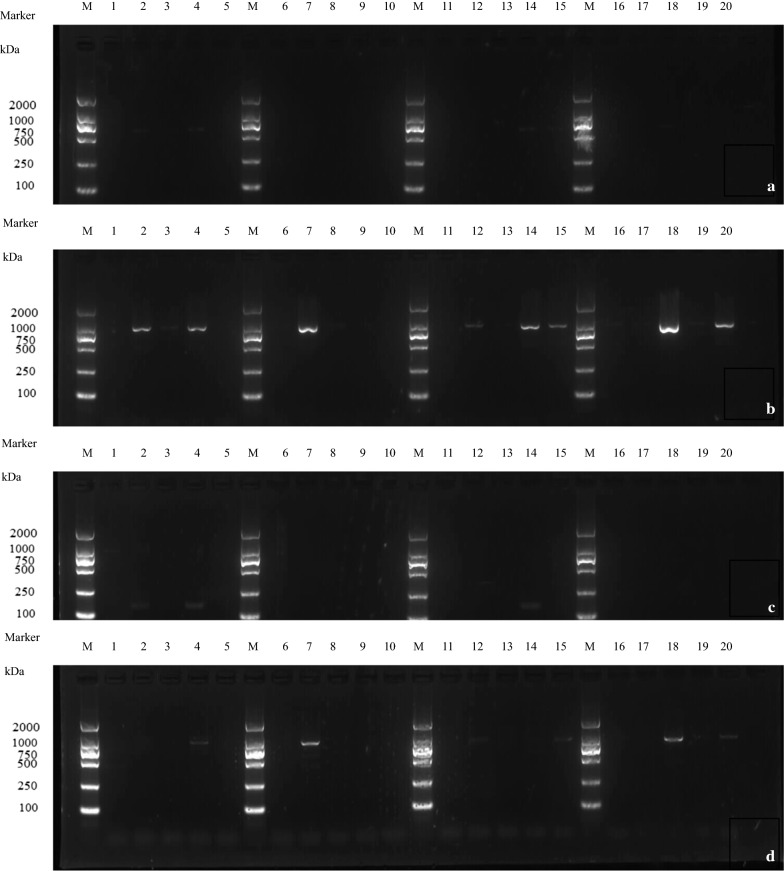
Effect of active compounds on expression of genes encoded PVY functional proteins of *VPg*, *CP*, *6K1*, and *P1*. **a** gene *VPg;* **b** gene *CP;* **c** gene *6K1;* **d** gene *P1.* M: Marker lane; Series of Lanes 1–5: compound **5**; 6–10: compound **1;** 11–15: compound **9**; 16–20: compound **4.** Each series of 5 lanes between markers represents the concentrations used: 500, 250, 125, 63, and 31 μg/mL, from left to right

The expressions of genes *HC*-*pro*, *P1*, *P3*, *NIa*, *NIb*, *6K1*, *VPg*, and *CP* under treatment with compounds **1**, **4**, **5**, and **9** at 31, 63, 125, 250, and 500 μg/mL were determined by PCR (Figs. 2 and 3).

As shown in Fig. 2, organizing data by gene, *HC*-*pro* and *NIb* showed no expression under treatment with compounds **1**, **4**, and **9** at all concentrations, and with compound **5** at 125 and 500 μg/mL (Fig. 2a, c). Gene *P3* showed no expression under treatment with compounds **1**, **4**, **5**, and **9** at all concentrations (Fig. 2b). Gene *NIa* showed no expression under treatment with compounds **1** and **4** at all tested concentrations, compound **5** at 125 and 500 μg/mL, and compound **9** at 125, 250, and 500 μg/mL (Fig. 2d). Organizing data by compound, at all tested concentrations: **1** and **4** significantly inhibited the expressions of genes *HC*-*pro*, *P3*, *NIb*, and *NIa*, compound **5** inhibited gene *P3*, and compound **9** inhibited *NIb*.

As shown in Fig. 3, with data organized by gene, genes *VPg* and *6K1* showed no expression under treatment with compounds **1**, **4**, **5**, and **9** at all tested concentrations (Fig. 3a, c). Gene *CP* showed no expression under treatment with compound **1** at 63, 125, and 500 μg/mL, compound **4** at 250 and 500 μg/mL, compound **5** at 125 and 500 μg/mL, and compound **9** at 125, 250, and 500 μg/mL (Fig. 3b). Gene *P1* showed no expression under treatment with compound **1** at 63, 125, and 500 μg/mL, compound **4** at 63, 250, and 500 μg/mL, compound **5** at 125, 250, and 500 μg/mL, and compound **9** at 63, 125, 250, and 500 μg/mL (Fig. 3d). Organizing the data by compound, compounds **1**, **4**, **5**, and **9** significantly inhibited the expressions of genes *VPg* and *6K1* at all tested concentrations.

### Effect of active compounds on the expression of early response genes induced by potato virus Y

#### Effects of active compounds on the expression of chlorophyll a-b binding protein (*Lhc*)

**Fig. 4:**
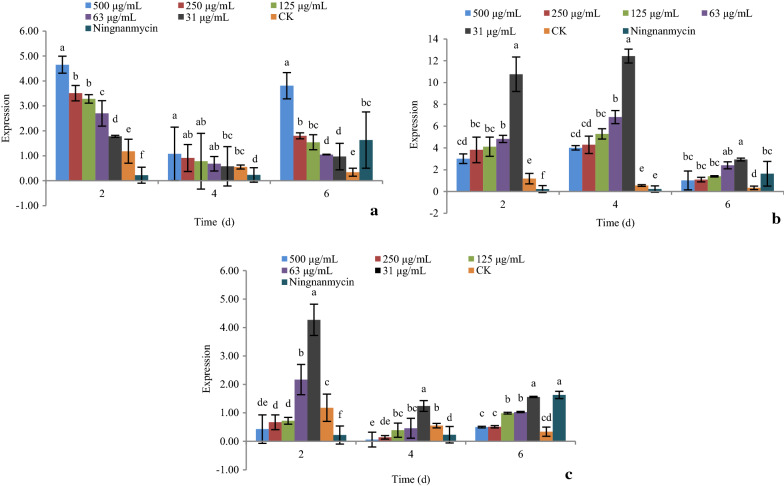
The effect of active compounds **1**, **5**, and **9** on the expression of gene *Lhc*. **a** Compound **1**; **b** Compound **5**; **c** Compound **9**

As shown in Fig. 4, the expression of gene *Lhc* under the treatment of compounds **1**, **5**, and **9** at 31, 63, 125, 250, and 500 μg/mL after 2, 4, and 6 days were determined by real-time PCR.

For compound **1**, increasing the concentration caused significant up-regulation of the gene *Lhc*. Over time, the gene was significantly down-regulated at day 4, then up-regulated at day 6, at all concentrations. The expression of gene *Lhc* was at its lowest at day 4 (Fig. 4a).

For compound **5**, increasing the concentration caused significant down-regulation of the gene *Lhc*. The gene was significantly up-regulated at day 4, and then down-regulated at day 6, at all concentrations. The expression of gene *Lhc* was at its highest at day 4 (Fig. 4b).

For compound **9**, increasing the concentration caused significant down-regulation of the gene *Lhc*. The gene was significantly down-regulated at day 4, and then up-regulated at day 6, at all concentrations. The expression of gene *Lhc* was at its lowest at day 4 (Fig. 4c).

#### Effect of active compounds on the expression of photo system II (*PSii*)

**Fig. 5:**
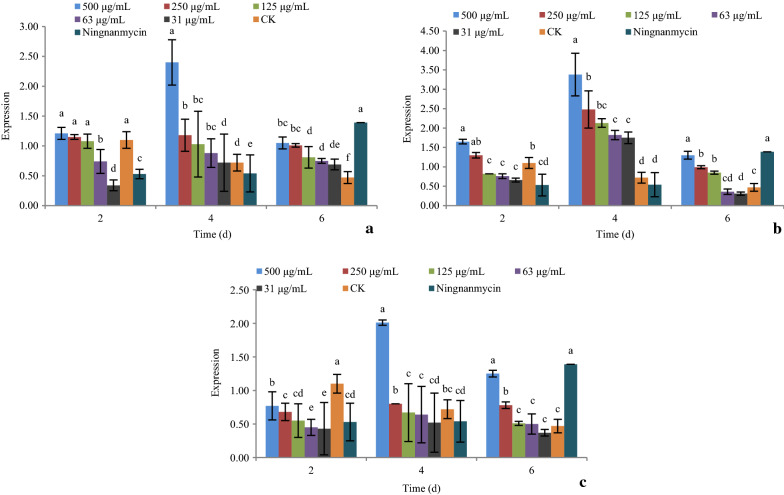
The effect of active compounds **1**, **5**, and **9** on the expression of gene *PSii*. **a** Compound **1**; **b** Compound **5**; **c** Compound **9**

As shown in Fig. 5, the expression of gene *PSii* under treatments with compounds **1**, **5**, and **9** at 31, 63, 125, 250, and 500 μg/mL after 2, 4, and 6 days was determined by real-time PCR.

For compounds **1**, **5**, and **9**, increasing the concentration caused significant up-regulation of the gene *PSii*. The gene was significantly up-regulated at day 4, then down-regulated at day 6, at all concentrations. The expression of gene *PSii* was at its highest at day 4.

#### Effect of active compounds on the expression of xyloglucan endotransglucosylase hydrolase (*XTH*)

**Fig. 6:**
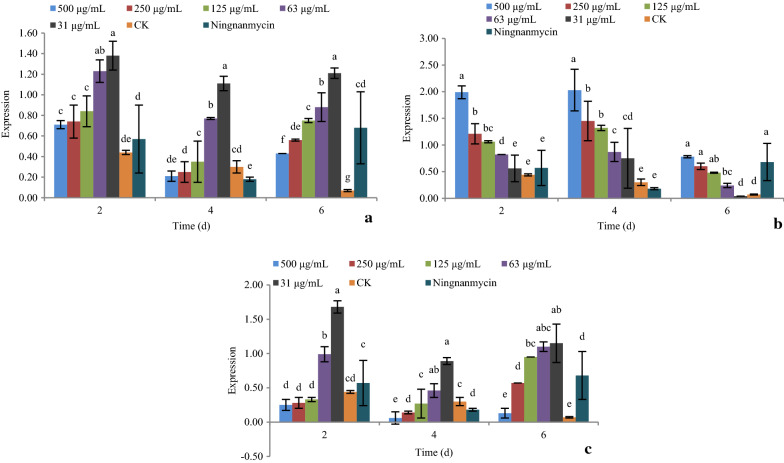
The effect of active compounds **1**, **5**, and **9** on the expression of gene *XTH*. **a** Compound **1**; **b** Compound **5**; **c** Compound **9**

As shown in Fig. 6, the expression of gene *XTH* under treatments with compounds **1**, **5**, and **9** at 31, 63, 125, 250, and 500 μg/mL after 2, 4, and 6 days was determined by real-time PCR.

For compound **1** and **9**, increasing the concentration caused significant down-regulation of the gene *XTH*. The gene was significantly down-regulated at day 4, and then up-regulated at day 6, at all concentrations. The expression of gene *XTH* was at its lowest at day 4 (Fig. 6a, c).

For compound **5,** increasing the concentration caused significant up-regulation of the gene *XTH*. The gene was significantly up-regulated at day 4, and then down-regulated at day 6, at all concentrations. The expression of gene *XTH* was at its highest at day 4 (Fig. 6b).

#### Effect of active compounds on the expression of gene CP-interacting protein 3 (*CPIP3*)

**Fig. 7:**
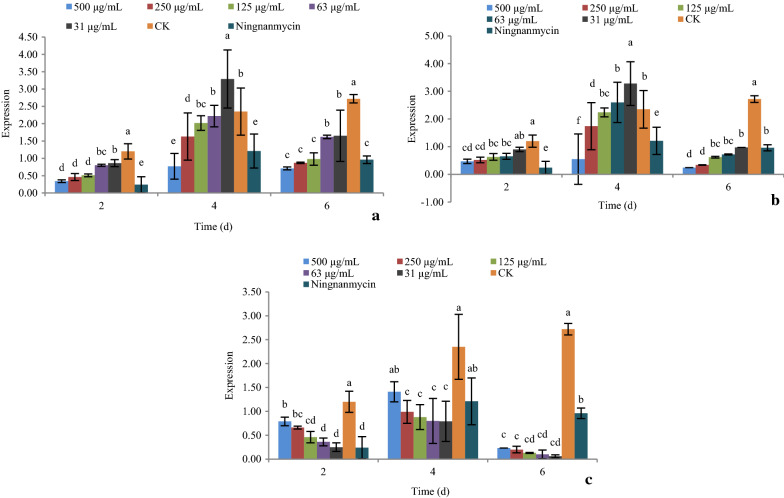
The effect of active compounds **1**, **5**, and **9** on the expression of gene *CPIP3*. **a** Compound **1**; **b** Compound **5**; **c** Compound **9**

As shown in Fig. 7, the expression of gene *CPIP3* under treatment with compounds **1**, **5**, and **9** at 31, 63, 125, 250, and 500 μg/mL after 2, 4, and 6 days was determined by real-time PCR.

For compounds **1** and **5**, increasing the concentration caused significant down-regulation of the gene *CPIP3*. The gene was significantly up-regulated at day 4, and then down-regulated at day 6, at all concentrations. The expression of gene *CPIP3* was at its highest at day 4 (Fig. 7a, b).

For compound **9**, increasing the concentration caused significant up-regulation of the gene *CPIP3*. The gene was significantly up-regulated at day 4, and then down-regulated at day 6, at all concentrations. The expression of gene *CPIP3* was at its highest at day 4 (Fig. 7c).

#### Effect of active compounds on the expression of the gene cullin-1 isoform (*cullin*-*1*)

**Fig. 8:**
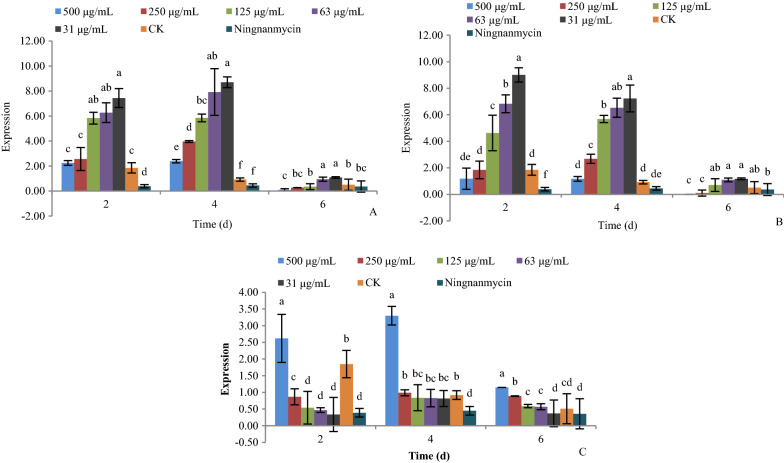
The effect of active compounds **1**, **5**, and **9** on the expression of gene *cullin*-*1*. **a** Compound **1**; **b** Compound **5**; **c** Compound **9**

As shown in Fig. 8, the expression of gene *cullin*-*1* under treatment with compounds **1**, **5**, and **9** at 31, 63, 125, 250, and 500 μg/mL after 2, 4, and 6 days was determined by real-time PCR.

For compound **1** and **5**, increasing the concentration caused significant down-regulation of the gene *cullin*-*1*. The gene was significantly up-regulated at day 4, and then down-regulated at day 6, at all concentrations. The expression of gene *cullin*-*1* was at its highest at day 4 (Fig. 8a, b).

For compound **9**, increasing the concentration caused significant up-regulation of the gene *cullin*-*1*. The gene was significantly up-regulated at day 4, and then down-regulated at day 6, at all concentrations. The expression of gene *cullin*-*1* was the highest at day 4 (Fig. 8c).

## Discussion

The genome of PVY is composed of a single-stranded RNA, length 9.7 kb, which covalently links with a viral-encoded protein (VPg) at its 5ʹ-end and contains a 3ʹ-polyadenylated tail. The genome of PVY encodes two polyproteins, a larger polyprotein of about 3000 amino acids and a shorter one translated from a 2^+^ frameshift in the P3 coding region. These polyproteins are cleaved by viral proteases, subsequently generating eleven mature proteins (Kim et al. 2015).

Compounds **1** and **4** significantly inhibited the expression of gene *HC*-*pro* at all concentrations tested. *HC*-*Pro* encodes viral helper component-proteinase (HC-Pro), which is a pathogen elicitor. It is also involved in multiple roles in aphid transmission, RNA binding, suppression of gene silencing and protease activity (Chowdhury et al. 2020; Kumar et al. 2020).

Compounds **1**, **4**, **5** and **9** significantly inhibited the expression of gene *VPg* at all concentrations tested. *VPg* encodes a viral protein called VPg which sabotages host antiviral RNA silencing to promote viral infection (Cheng et al. 2016).

Compounds **1**, **4**, **5**, and **9** significantly inhibited the expression of genes *CP* and *P1,* at some concentrations. *CP* encodes a viral coat protein and *P1* encodes plasma membrane cation-binding protein 1 (PCaP1), which is shown to be important for the intra-cellular movement of two members of the genus *Potyvirus*, in *Arabidopsis* (rockcress) and in tobacco plants (Beris et al. 2020).

Compounds **1** and **4** significantly inhibited the expression of gene *NIa* at all concentrations tested. *NIa* encodes the “nuclear inclusion a” (NIa) protease of PVY, which is involved in processing the polyprotein encoded by its positive sense RNA genome. The NIa protein is also responsible for the release of the VPg protein involved in viral RNA replication (Gargouri-Bouzid et al. 2006).

Compounds **1**, **4** and **9** significantly inhibited the expression of gene *NIb* at all concentrations tested. *NIb* is an RNA-dependent RNA polymerase in the PVY genome, which has been reported to confer virulence in infections of pepper plants (Janzac et al. 2010).

Compounds **1**, **4** and **5** significantly inhibited the expression of gene *P3* at all concentrations tested. *P3* encodes potyviral membrane protein.

Compounds **1**, **4**, **5**, and **9** significantly inhibited the expression of gene *6K1*. The function of *6K1* is currently unknown. In summary, all compounds showed inhibitory activities against various genes encoding functional proteins of PVY. The results provide a starting point for future research into the inhibitory mechanisms and pathways of active compounds against PVY.

The *Lhc* gene encodes chlorophyll a-b binding protein, which takes part in regulating photosynthesis and chlorophyll synthesis. The down-regulation of this gene means a decrease in viral nucleic acid, which is also seen in PVY-resistant plants (Chen et al. 2017). Compounds **5** and **9** down-regulated the *Lhc* gene. There was an inverse relationship between concentration and gene expression. At day 4, the expression of gene *Lhc* was at its lowest. These results suggest that compounds **5** and **9** elicit their inhibitory activity due to their influence on chlorophyll synthesis and photosynthesis during days 2−4.

The *PSii* gene takes part in photosystem II of photosynthesis. A down-regulation of this gene has the same effect as inhibition of *Lhc*: a decrease in viral nucleic acid. Compounds **1**, **5**, and **9** showed no significant inhibitory effect of this gene, but the expression of *PSii* gene was at its lowest on day 6.

The *XTH* gene encodes xyloglucan endotransglucosylase hydrolase, which is an essential constituent of the primary cell wall and participates in cell wall elongation and construction (Otulak-Kozieł K et al. 2018a; Otulak-Kozieł K et al. 2018b). The cell wall is the first barrier that protects plants against entry of pathogens and other harmful bodies, and offers protection against mechanical stress to plant cells. The expression of cell-wall-related genes, including *XTH,* has been shown to be regulated by biotic and abiotic stresses (Li et al. 2005). In this study, *XTH* was up-regulated under treatment with compound **5** as the concentration increased before 4 days. This suggests that compound **5** could increase elongation and construction of plant cell walls at different concentrations, especially at day 4. However, the *XTH* gene showed down-regulation under treatment with compounds **1** and **9** at day 4 and then up-regulation at day 6. This suggests that both compounds **1** and **9** could in increase elongation and construction of plant cell walls after 4 days, especially at day 6. It affected the plant cell wall later than that of compound **5**. This may indicate that these compounds contribute to induce PVY resistance in *Nicotiana.*

*CPIP3* is a stress response gene, encoding CP-interacting protein 3. The interaction between viral coat protein (CP) and host plant CP-interacting protein were shown to be important for the plant defense response, viral propagation, and long distance movement (Li et al. 2005; Park et al. 2009). A low expression of the *CPIP3* gene signifies a low quantity of CP-interacting protein 3 which, to some extent, signifies inhibitory activity against PVY (Li et al. 2005). In this study, compounds **1** and **5** significantly down-regulated the gene *CPIP3* at 2 days, with their increased concentration. Therefore, compounds **1** and **5** could inhibit the viral multiplication of PVY by inducing the plant defense response in tobacco plants. Although compound **9** was able to up-regulate *CPIP3* at days 2 and 4, by day 6 there was significant down-regulation of the gene, at all concentrations tested. The result demonstrated the inhibitory activity of compound **9** against the multiplication of PVY, by inducing the tobacco plant defense response at 6 days.

Gene *cullin*-*1* also encodes a stress response protein. The up-regulation of it was reported to be important in the resistance response to pathogen infection and autoimmunity (Cheng et al. 2011; Gou et al. 2009; Liu et al. 2002). In our study, the gene *cullin*-*1* was significantly up-regulated by compounds **1**, **5,** and **9** at days 2 and 4. This meant that all three compounds had significant inhibitory activity against PVY by inducing the resistance response to PVY infection and autoimmunity in tobacco plants at 2–4 days. In detail, compounds **1** and **5** up-regulated *cullin*-*1* at low concentrations of 31, 63, and 125 μg/mL, while compound **9** up-regulated *cullin*-*1* at a high concentration of 500 μg/mL.

Taken together, active compounds **1**, **5**, and **9** had significant inhibitory activity against PVY and various effects on the expression of viral genes related to pathogenesis. They might be considered as potential antiviral agents for future development.

## Supplementary information

**Additional file 1.** The data of ^1^H NMR and ^13^C NMR of compounds **1**–**9**

## Data Availability

We admit availability of data and material.
