# Supplementary material for: The inhibitory effects of metabolites from Bacillus pumilus on potato virus Y and the induction of early response genes in Nicotiana tabacum
Source: AMB Express. 2020 Aug 20;10:152. doi: 10.1186/s13568-020-01089-1 (PMC7441133; doi:10.1186/s13568-020-01089-1)
Supplement: Supplementary file 1 — Additional file 1. The data of 1H NMR and 13C NMR of compounds 1–9 [file 13568_2020_1089_MOESM1_ESM.docx]

**AMB EXPRESS**

**The inhibitory effects of metabolites from *Bacillus pumilus* on potato virus Y and the induction of early response genes in *Nicotiana tabacum***

Shuo Shen^1, 2, 3, 4^ · Wei Li ^2, 3, 4*^

^1^ Guizhou University, Qinghai University, Guiyang 550025, Guizhou, China

^2^ Key Laboratory of Potato Breeding of Qinghai Province, Xining 810016, Qinghai, China

^3^ State Key Laboratory of Plateau Ecology and Agriculture, Xining 810016, Qinghai, China

^4^ The Tibet Plateau Biotechnology Key Lab of Ministry of Education, Xining 810016, Qinghai, China

*Correspondence: Wei Li; E-mail: lwbabylw@163.com; Tel: +86-971-5312130; Fax: +86-971-5312130.

**The data of ^1^H NMR and ^13^C NMR of compounds 1–9**

*Cyclo(L-Leu-L-Pro)* (**1**). ^1^H NMR (600 MHz, methanol-*d*_4_) δ 4.32 (*t*, 1H, *J* = 7.9 Hz, H-5), 4.18 (*dd*, 1H, *J* = 8.0, 4.1 Hz, H-2), 3.54 (*dd*, 2H, *J* = 8.7, 5.0 Hz, H_2_-8), 2.33 (*m*, 1H, H_2_-6), 2.10-1.86 (*overlap*, 4H, H_2_-7, H-2´), 1.56 (*td*, 1H, *J* = 7.7, 3.6 Hz, H_2_-1´), 0.99 (*overlap*, 6H, H_3_-4´); ^13^C NMR (150 MHz, methanol-*d*_4_) δ 22.2 (C-3´), 23.3 (C-4´), 23.6 (C-6), 25.7 (C-2´), 29.0 (C-7), 39.3 (C-1´), 46.4 (C-8), 54.5 (C-2), 60.2 (C-5), 168.8 (C-1), 172.8 (C-4).

*Cyclo(L-Pro-L-Tyr)* (**2**). ^1^H NMR (600 MHz, methanol-*d*_4_) δ 7.01 (*d*, 2H, *J* = 8.5 Hz,), 6.68 (*d*, 2H, *J* = 8.5 Hz,), 4.33 (*td*, 1H, *J* = 4.9, 1.9 Hz,), 4.01 (*ddd*, 1H, *J* = 11.0, 6.3, 2.0 Hz,), 3.51 (*dt*, 1H, *J* = 12.0, 8.3 Hz,), 3.32 (*dt*, 1H, *J* = 12.4, 6.5 Hz,), 3.08 - 2.97 (*m*, 2H), 2.06 (*m*, 1H), 1.77 (*m*, 2H), 1.20 (*m*, 1H). ^13^C NMR (151 MHz, methanol-*d*_4_) δ 22.7 (C-4), 29.4 (C-5), 37.6 (C-10), 45.9 (C-3), 57.9 (C-9), 60.0 (C-6), 116.2 (C-3´, 5´), 127.6 (C-1´), 132.1 (C-2´, 6´), 157.7 (C-4´), 166.9 (C-1), 170.8 (C-7).

*Brevianamide F* (**3**). ^1^H NMR (600 MHz, Methanol-*d*_4_) δ 7.56 (*d*, 1H, *J* = 8.0 Hz, H-5´), 7.32 (*d*, 1H, *J* = 8.1 Hz, H-8´), 7.10-7.06 (*overlap*, 2H, H-3´, 6´), 7.00 (*ddd*, 1H, *J* = 8.1, 6.9, 1.0 Hz, H-7´), 4.39 (*td*, 1H, *J* = 5.1, 1.8 Hz, H-2), 3.96 (*ddd*, 1H, *J* = 10.8, 6.4, 1.9 Hz, H-4), 3.44 (*dt*, 1H, *J* = 11.8, 8.2 Hz, H-1´), 3.24 (*ddd*, 1H, *J* = 11.8, 9.5, 3.8 Hz, H-7_b_), 1.95 (*m*, 1H, H-5_a_), 1.66 (*m*, 1H, H-6_a_), 1.46 (*m*, 1H, H-5_b_), 0.95 (*m*, 1H, H-6_b_). ^13^C NMR (151 MHz, methanol-*d*_4_) δ 170.7 (C-3), 167.4 (C-1), 138.0 (C-9´), 128.7 (C-4´), 125.6 (C-3´), 122.6 (C-7´), 119.9 (C-6´), 119.8 (C-5´), 112.3 (C-8´), 109.5 (C-2´), 60.0 (C-4), 57.2 (C-2), 45.9 (C-7), 29.1 (C-5), 29.1 (C-1´), 22.5 (C-6).

*2-(3-indolyl) ethanol* (**4**). ^1^H NMR (600 MHz, methanol-*d*_4_) δ 7.51 (*d*, 1H, *J* = 7.8 Hz, H-4), 7.30 (*d*, 1H, *J* = 7.8 Hz, H-7), 7.05 (*ddd*, 1H, *J* = 1.0, 6.9, 8.0 Hz, H-2), 7.03 (*s*, 1H, *J* = 7.8 Hz, H-6), 6.97 (*ddd*, 1H, *J* = 1.0, 6.9, 8.0 Hz, H-5), 3.79 (*t*, 2H, *J* = 7.3 Hz, H-11), 2.95 (*t*, 2H, *J* = 7.3 Hz, H-10), 9.97 (*s*, 1H, 1-NH). ^13^C NMR (150 MHz, MeOD) δ 29.8 (C-10), 63.7 (C-11), 112.1 (C-7), 112.7 (C-3), 119.2 (C-6), 119.5 (C-4), 122.2 (C-5), 123.5 (C-2), 128.9 (C-9), 138.1 (C-8).

*N-[2-(1H-indol-3-yl) ethyl] acetamide* (**5**). ^1^H NMR (600 MHz, Methanol-*d*_4_) δ 7.52 (*d*, 1H, *J* = 7.9 Hz, H-4), 7.30 (*d*, 1H, *J* = 8.2 Hz, H-1), 7.06 (*m*, 2H, *J* = 7.7 Hz, H-3), 7.01 (*s*, 1H, H-7), 6.98 (*m*, 1H, *J* = 7.5 Hz, H-2), 3.42 (*t*, 3H, *J* = 7.4 Hz, H-9), 2.89 (*td*, 3H, *J* = 7.4, 0.9 Hz, H-10), 1.86 (*s*, 3H, H-12). ^13^C NMR (151 MHz, methanol-*d*_4_) δ 22.6 (C-12), 26.2 (C-9), 41.5 (C-10), 112.2 (C-1), 113.2 (C-8), 119.2 (C-4), 119.6 (C-3), 122.3 (C-2), 123.3 (C-7), 128.7 (C-5), 138.1 (C-6), 173.2 (C-11).

*3, 3-di(1H-indol-3-yl)propane- 1,2-diol* (**6**). ^1^H NMR (600 MHz, methanol-*d*_4_) δ 7.55 (*m*, 2H, *J* = 7.9, 4.7 Hz, H-6´), 7.29 (*overlap*, 3H, *J* = 8.0 Hz, H-7´, 7´´), 7.13 (*s*, 1H, H-2´´), 7.02 (*m*, 2H, *J* = 7.0 Hz, H-4´), 6.91 (*m*, 2H, H-5´), 4.69 (*d*, 1H, *J* = 6.6 Hz, H-3), 4.49 (*td*, 1H, *J* = 6.9, 4.0 Hz, H-2), 3.61 (*dd*, 1H, *J* = 11.2, 4.0 Hz, H_a_-1), 3.49 (*dd*, 1H, *J* = 11.1, 7.2 Hz, H_b_-1). ^13^C NMR (151 MHz, methanol-*d*_4_) δ 38.1 (C-3), 66.4 (C-1), 76.4 (C-2), 112.0 (C-7´), 112.1 (C-7´´), 116.4 (C-3´), 118.0 (C-3´´), 119.3 (C-5´), 119.4 (C-5´´), 120.0 (C-4´), 120.2 (C-4´´), 122.0 (C-6´), 122.1 (C-6´´), 123.9 (C-2´), 124.1 (C-2´´), 128.4 (C-4´a), 129.3 (C-4´´a), 138.0 (C-7´a), 138.1 (C-7´´a).

*Lincomycin B* (**7**). ^1^H NMR (600 MHz, methanol-*d*_4_) δ 5.26 (d, *J* = 5.6 Hz, 1H), 4.41 (dd, *J* = 9.1, 4.9 Hz, 1H), 4.29 (dd, *J* = 9.5, 6.2 Hz, 1H), 4.19 (d, *J* = 9.1 Hz, 1H), 4.10 (ddd, *J* = 11.4, 6.0, 4.4 Hz, 2H), 3.92 (d, *J* = 3.4 Hz, 1H), 3.80 – 3.74 (m, 1H), 3.56 (dd, *J* = 10.2, 3.3 Hz, 1H), 2.94 (s, 3H), 2.93 – 2.86 (m, 1H), 2.31 – 2.24 (m, 5H), 2.11 (s, 3H), 2.03 (s, 1H), 1.57 – 1.50 (m, 2H), 1.18 (d, *J* = 6.4 Hz, 4H), 0.96 (t, *J* = 7.4 Hz, 3H). ^13^C NMR (150 MHz, methanol-*d*_4_) δ 12.5, 13.8, 17.7, 26.6, 36.3, 39.8, 41.0, 55.7, 62.1, 67.6, 69.4, 69.7, 70.2, 70.6, 72.0, 90.2, 169.5.

*Dibutylphthalate* (**8**). ^1^H NMR (600 MHz, methanol-*d*_4_) δ 7.71 (*dd*, 2H, *J* = 3.4, 5.7 Hz, H-3, 6), 7.60 (*dd*, 2H, *J* = 3.4, 5.7 Hz, H-4, 5), 4.28 (*t*, 4H, *J* = 6.6 Hz, H-8, 8´), 1.71 (*m*, 4H, *J* = 6.6 Hz, H-9, 9´), 1.45 (*m*, 4H, *J* = 7.4 Hz, H-10, 10´), 0.97 (*t*, 6H, *J* = 7.4 Hz, H-11, 11´). ^13^C NMR (150 MHz, MeOD) δ 14.1 (C-11, 11´), 20.3 (C-10, 10´), 31.7 (C-9, 9´), 66.6 (C-8, 8´), 129.9 (C-3, 6), 132.3 (C-4, 5), 133.6 (C-1, 2), 169.3 (C-7, 7´).

*P-hydroxyphenethyl alcohol* (**9**). ^1^H NMR (600 MHz, methanol-*d*_4_) δ 7.01 (*d*, 2H, *J* = 8.5 Hz, H-2, 6), 6.68 (*d*, 2H, *J* = 8.4 Hz, H-3, 5), 3.67 (*t*, 2H, *J* = 7.2 Hz, H-8), 2.70 (*t*, 2H, *J* = 7.2 Hz, H-7). ^13^C NMR (150 MHz, methanol-*d*_4_) δ 39.4 (C-7), 64.6 (C-8), 116.1 (C-3, 5), 130.9 (C-2, 6), 131.0 (C-1), 156.8(C-4).
